# Effects of prolonged oxytetracycline supplementation on freshwater stinging catfish (*Heteropneustes fossilis*): a multi-biomarker approach

**DOI:** 10.3389/fimmu.2024.1478114

**Published:** 2024-12-19

**Authors:** Pushpa Choudhary, Saisweta P. Naik, Sameer Ranjan Sahoo, Rakesh Das, Satya Narayan Sahoo, Satyen Kumar Panda, Thangapalam Jawahar Abraham, Prasanna Kumar Patil, Priyabrat Swain, Sudhansu Sekhar Mishra

**Affiliations:** ^1^ Indian Council of Agriculture Research (ICAR)-Central Institute of Freshwater Aquaculture (CIFA), Fish Health Management Division, Bhubaneswar, Odisha, India; ^2^ Fish Processing Division, Indian Council of Agriculture Research (ICAR)-Central Institute of Fisheries Technology, Cochin, Kerala, India; ^3^ Department of Aquatic Animal Health, Faculty of Fishery Sciences, West Bengal University of Animal and Fishery Sciences, Kolkata, West Bengal, India; ^4^ Indian Council of Agricultural Research (ICAR)-Central Institute of Brackishwater Aquaculture (CIBA), Aquatic Animal Health and Environment Division, Chennai, Tamil Nadu, India

**Keywords:** aquaculture, *Heteropneustes fossilis*, oxytetracycline hydrochloride, immunological indices, biochemical parameters, tissue level retention

## Abstract

**Background:**

Aquaculture systems that sporadically depend on antibiotics can contribute to the development of adverse effects on the fish, microbial flora and the environment. This study sought to investigate the impacts of extended oxytetracycline supplementation on the freshwater stinging catfish *Heteropneustes fossilis* through a multi-biomarker approach.

**Methods:**

A total of 300 *H. fossilis* (20 ± 0.5 g) were placed in fibreglass-reinforced plastic tanks. The experimental fish were administered oxytetracycline hydrochloride (OTC) at varying doses, viz., 80 mg/kg fish biomass/day (1x), 240 mg (3x), 400 mg (5x), and 800 mg (10x) for 30 consecutive days. The study also included a control group that did not receive OTC.

**Results:**

OTC was effective against *Aeromonas hydrophila*, *Pseudomonas putida*, and *Plesiomonas shigelloides*, with minimum inhibitory concentrations ranging between 0.5 and 8.0 µg/mL. The OTC supplementation retarded the growth of fish. The respiratory burst activity, myeloperoxidase, and lysozyme increased significantly in the 1x group until day 20. This group showed an increase in serum albumin, whereas other OTC groups exhibited elevated levels of liver functional enzymes, including alanine transaminase, aspartate aminotransferase, and alkaline phosphatase. In addition, OTC groups exhibited increased levels of antioxidant enzymes. The magnitude of the increase was dose- and time-dependent. The liquid chromatography-mass spectrometry/mass spectrometry (LC-MS/MS) study signified a dose-dependent increase in OTC residues in the muscle. After a 10-day discontinuation of OTC, the tissue level retention of residues was minimal in the muscle, specifically in the 1x group compared to other groups. Significant histological alterations were noted in the liver tissues of the 5x and 10x groups, possibly due to oxidative stress and residue accretion.

**Conclusion:**

The therapeutic dose of 80 mg/kg biomass/day was safe and tolerated well by *H. fossilis*, and may be used for sustainable catfish farming practices.

## Highlights

- Effect of oxytetracycline (OTC) on *Heteropneustes fossilis* was evaluated
- OTC caused dose- and time-dependent changes in immune and biochemical parameters
- OTC effectively inhibited the bacterial fish pathogens
- Higher OTC doses caused oxidative stress and impaired liver tissue histoarchitecture
- LC-MS/MS analysis showed dose-dependent OTC accumulation in edible muscle

## Introduction

More innovative practices have replaced extensive traditional aquaculture practices in recent decades. These practices involve raising aquatic animals at high stocking densities, which increases the crowding stress and lowers immunity in reared animals. This, in turn, increases the risk of the emergence of new diseases, leading to financial difficulties and unsustainable fish farming (1). Among numerous devastating emerging fish diseases, bacterial infections are one of the important culprits for successful aquafarming (2). Ideally, fish farmers treat bacterial pathogens through medicated feed supplemented with desired levels of antibiotics for a prescribed period (3–5). Fish farmers often apply high doses of antibiotics for extended duration due to a lack of awareness on the impact of antibiotics (3), hence such a practice jeopardizes the safety of reared animals and end consumers. This injudicious use of antibiotics affects international trade and the aquatic environments (6). Research on the antibiotics that stay in fish muscle after long-term administration to treat bacterial diseases is the most important thing that necessitates the safe and wise use of antibiotics and the safety of aquatic products.

Oxytetracycline (OTC), an important antibiotic from the tetracycline group authorized for aquaculture use, is effective against the treatment of numerous bacterial pathogens (7), which works on the principle of inhibition of protein synthesis for bacterial lysis (8). However, studies have shown that it can hurt the fish liver and kidneys, change the microbiome of the gut, damage mitochondria, and make membrane lipid peroxidation worse (9, 10). Therefore, ensuring the biosafety of OTC in cultured aquatic animals is critical before advocating its widespread use. Environmental conditions significantly influence the efficacy of antimicrobials (11), with factors such as water temperature affecting fish metabolism due to their poikilothermic nature. Compliance with regional antimicrobial regulations, drug depletion studies, and dose-response analyses under specific environmental conditions are essential. Despite established guidelines for temperate fish species, there is a lack of information on OTC safety, efficacy, and residue accumulation in tropical regions (11). Recent studies have looked at how safe OTC is for fish species like rohu (12), snubnose pompano (13), Nile tilapia (14), striped catfish (15), mrigal (16), and catla (17).

Asian stinging catfish *Heteropneustes fossilis* is among the most extensively farmed freshwater omnivorous catfish of the Heteropneustidae family. Highly valued for its culinary appeal, low-fat content, high protein content, and medicinal properties (18, 19), this species thrives in challenging environments such as muddy waters with low oxygen levels. Its ability to tolerate prolonged periods of low oxygen and high ammonia concentrations further enhances its suitability for aquaculture, including stagnant water bodies like swamps and wetlands (20). To date, no study has investigated the impact of prolonged use of oxytetracycline hydrochloride (OTC) at gradually increasing doses through feed in *H. fossilis*. This study aimed to conduct a 30-day indoor in-feed OTC feeding trial at varying doses ranging from the therapeutic dose of OTC (1x; 80 mg/kg fish biomass/day) to 10 times the therapeutic dose (10x: 800 mg/kg fish biomass/day) and to explore the effects of OTC on non-specific immunity, serum biochemistry, oxidative stress biomarkers, residue retention in the edible muscle and liver histopathological alterations. Besides, the minimum inhibitory concentration (MIC) of OTC against common bacterial fish pathogens was determined to develop appropriate strategies on the dose and dosage, ensuring its sparing and responsible use in catfish aquaculture.

## Materials and method

### Animal ethics

Experimental trials were carried out in the wet laboratory at the Fish Health Management Division of the ICAR-Central Institute of Freshwater Aquaculture, (ICAR-CIFA) Bhubaneswar, India. The experiments were completed under the guidelines provided in ARRIVE (21) and the ethical committee of ICAR-CIFA, Bhubaneswar. All the methods and procedures of the All-India Network Project on Fish Health were endorsed by the Indian Council of Agricultural Research, Government of India, New Delhi (CIBA/AI-NPFH/2020-2025). Every procedure was as per the humane condition to reduce any sort of trouble to the experimental animals.

### Determination of minimum inhibitory concentration of OTC against fish pathogens

The MIC of OTC against 8 fish pathogenic bacterial strains comprising four *Aeromonas hydrophila* and two each of *Pseudomonas putida* and *Plesiomonas shigelloides* was evaluated following the microplate broth dilution method (22).

### Experimental conditions, setup and medicated feed preparation

A total of 300 stinging catfish devoid of any infections were sourced from local farmers near ICAR-CIFA, Kausalyaganga, Bhubaneswar, and acclimatized for three weeks in 1000 L fibreglass-reinforced plastic (FRP) tanks. The incorporation of OTC (Sigma-Aldrich, USA) into the basal diet was based on fish biomass as per the therapeutic dose of 80 mg/kg biomass/day (7). The experimental feeding trial included four treatment groups, in triplicates, with 20 fish in each tank, i.e., 1x (80 mg), 3x (240 mg), 5x (400 mg), and 10x (800 mg), as well as a control group with no OTC. The feed formulation involved essential nutrient inclusion with a basic protein content of 300 g/kg for the basal feed. OTC was added according to treatment specifications. The ingredients were accurately weighed, mixed, and pressure-cooked for 20 minutes to improve palatability. Afterwards, thoroughly blended the mineral and vitamin mix and the required amount of OTC were mixed with the cooked dough (**Table 1**). A hand pelletizer was used to shape the dough into 2 mm feed crumbles, dried in a hot air oven at 40°C for 48 h. The dried crumbles were stored in a zip lock polythene pouch at 4°C. The experiment spanned 40 days comprised of 30 days of OTC administration at graded doses as above and 10 days of post-OTC-administration. The fish groups were fed appropriate feeds daily at 3% body weight twice daily in equal rations. The water quality parameters such as temperature (28.5 - 29°C), pH (7.8 - 8.4), dissolved oxygen (5.1 - 5.7 mg/L), total alkalinity (121 - 134 mg/L), total ammonia nitrogen (0.44 - 0.87 mg/L), nitrate (0.75 - 0.95 mg/L, and nitrite (0.31 - 0.85 mg/L) were maintained optimally throughout the experiment.

**Table 1:** Composition of medicated feed (gram/kg feed).

Ingredients	Control (Basal diet)	T1 (80 mg/kg biomass/day)	T2 (240 mg/kg biomass/day)	T3 (400 mg/kg biomass/day)	T4 (800 mg/kg biomass/day)
Fish meal	50	50	50	50	50
Soya meal	280	280	280	280	280
Ground nut oil cake	260	260	260	260	260
Rice bran	200	200	200	200	200
Corn flour	150	147.34	142	136.7	123.4
Oil	40	40	40	40	40
Vitamin and mineral mix	20	20	20	20	20
OTC	**0**	**2.66**	**8.0**	**13.3**	**26.6**

OTC, Oxytetracycline hydrochloride.* As in case of T1, 1 kg biomass was provided with 30 gm of medicated feed (3% BW/day), that contained 80 mg OTC.

### Blood, serum and liver tissue collection

Every ten days, six fish were randomly picked from each treatment group (2 fish/tank) for the collection of blood, serum, edible muscle and liver tissues. Before collecting blood, 50 µL/L of clove oil was applied as a fish sedative. Blood was collected from the caudal peduncle using a single-use 2.0 mL syringe and a portion was transferred to EDTA-coated vials for the assessment of respiratory burst activity (RBA). To separate serum, the entire blood without anticoagulant was held in a 1.5 mL microcentrifuge tube in a slanting position for one hour followed by 4 hours of incubation at 4°C. The serum was separated by centrifugation at 3000 g for 10 min in a refrigerated centrifuge (Multifuge X1R, Thermo Scientific, USA) and stored at -20°C until use. The humanely euthanized fish using clove oil (100 µL/L) was carefully dissected, to remove the liver of each fish, The liver tissues were blended using a homogenizer (Qiagen Hilden, Germany) set to run at 8000 rpm for 30 sec with chilled 0.05 M tris-hydrochloride (pH: 8.0). The homogenate was centrifuged at 13,000 g at 4°C for 15 min to extract tissue fragments and lipids, transferred to the 1.5 mL Eppendorf vials and stored at -20°C until the enzymatic testing.

### Immunological and serum biochemical parameters

Blood RBA and serum myeloperoxidase (MPO) were assessed as previously described (23, 24). The serum lysozyme was determined by following the (25) method. Serum albumin was measured by bromocresol green binding using an albumin kit (Coral Clinical Systems, India). The serum glucose, alanine transaminase (ALT), aspartate aminotransferase (AST), and alkaline phosphatase (ALP) were estimated according to the kit’s instructions manufactured by Coral Clinical Systems, India.

### Oxidative stress biomarkers in liver tissue

The activity of liver SOD was measured based on a cytochrome C reduction test utilizing a commercial kit (Cayman Chemicals, India), with absorbance obtained at 550 nm (26). CAT activity was evaluated using a commercial kit to break down hydrogen peroxide (27). Malondialdehyde (MDA) was assessed using a colourimetric approach based on the thiobarbituric acid reaction, calculated using commercial kits (Cayman Chemicals, India) as previously described (26)

### Analysis of oxytetracycline residues in edible muscle tissue

To quantify OTC residues in edible muscle tissue, a brief modification of the method as illustrated (12) was employed. Approximately 15 g of pooled tissue from two fish/tank on days 10, 20, and 30 of OTC-dosing, and day 10 post-OTC-dosing (40^th^ day of the experiment) were individually homogenized. Each homogenate (approx. 5.0-6.0 g) was mixed in a 50 mL falcon tube containing 20 mL of acetone and centrifuged at 8000 g for 15 min, resulting supernatant was transferred to a 100 mL round bottom flask and subjected to a second extraction with 10 mL of acetone. A rotary evaporator (Heidolph Laborota 4000, Germany) was used for concentrating total supernatants for removing acetone so that an aqueous solution could be obtained. The aqueous eluate was then chromatographically separated and collected. Following evaporation with a rotary evaporator, the eluate was dissolved in 2 mL of acetonitrile containing 1% acetic acid. Subsequently, 5 μL of the resulting solution was injected into an LC-MS/MS instrument for quantification of OTC, which exhibited a retention time of approximately 5 min.

### Liver histopathology

On days 10, 20, 30, and 40 of the experiment, a portion of liver tissues from each fish (six fish/group) was preserved in 10% neutral buffered formalin, followed by tissue processing and paraffin wax embedding. Wax blocks were subjected to a microtoming process to create 5.0 µm tissue sections, thereafter stained with haematoxylin and eosin (28), and observed under a microscope (Olympus, Japan) for documentation of tissue alterations.

### Statistical analysis

All the data were analyzed using the statistical package (SPSS Inc, USA, IBM) and represented as mean with standard deviation. The homogeneity of variances in the data was checked using Levene’s test. One-way analysis of variance and Duncan’s multiple range test were used to determine the significance at P<0.05 among treatments and control.

## Results

### MIC of OTC against fish pathogens

The MICs of OTC against common freshwater fish pathogenic strains such as *A. hydrophila P. putida* and *P. shigelloides* were in the range of 0.5–8.0 µg/mL (**Table 2**).

**Table 2:** Minimal inhibitory concentration (MIC) of oxytetracycline against fish pathogenic bacterial strains.

Bacterial species	Strain code	Tissue source	Fish species	Year of isolation	MIC (μg/mL)
*Aeromonas hydrophila*	CIFA/AHy-03	Kidney	*Labeo rohita*	2021	0.5
	CIFA/AHy-07	Blood	*Anabas testudineus*	2019	2.0
	CIFA/AHy-14	Ulcer	*Catla catla*	2020	2.0
	CIFA/AHy-15	Kidney	*H. fossilis*	2023	1.0
*Pseudomonas putida*	CIFA/PPu-05	Blood	*H. fossilis*	2022	0.5
	CIFA/PPu-15	Liver	*H. fossilis*	2022	8.0
*Plesiomonas shigeloides*	CIFA/PSh-01	Kidney	*H. fossilis*	2023	2.0
	CIFA/PSh-06	Blood	*H. fossilis*	2023	1.0

### Fish biomass and non-specific immunity

**Figure 1:**
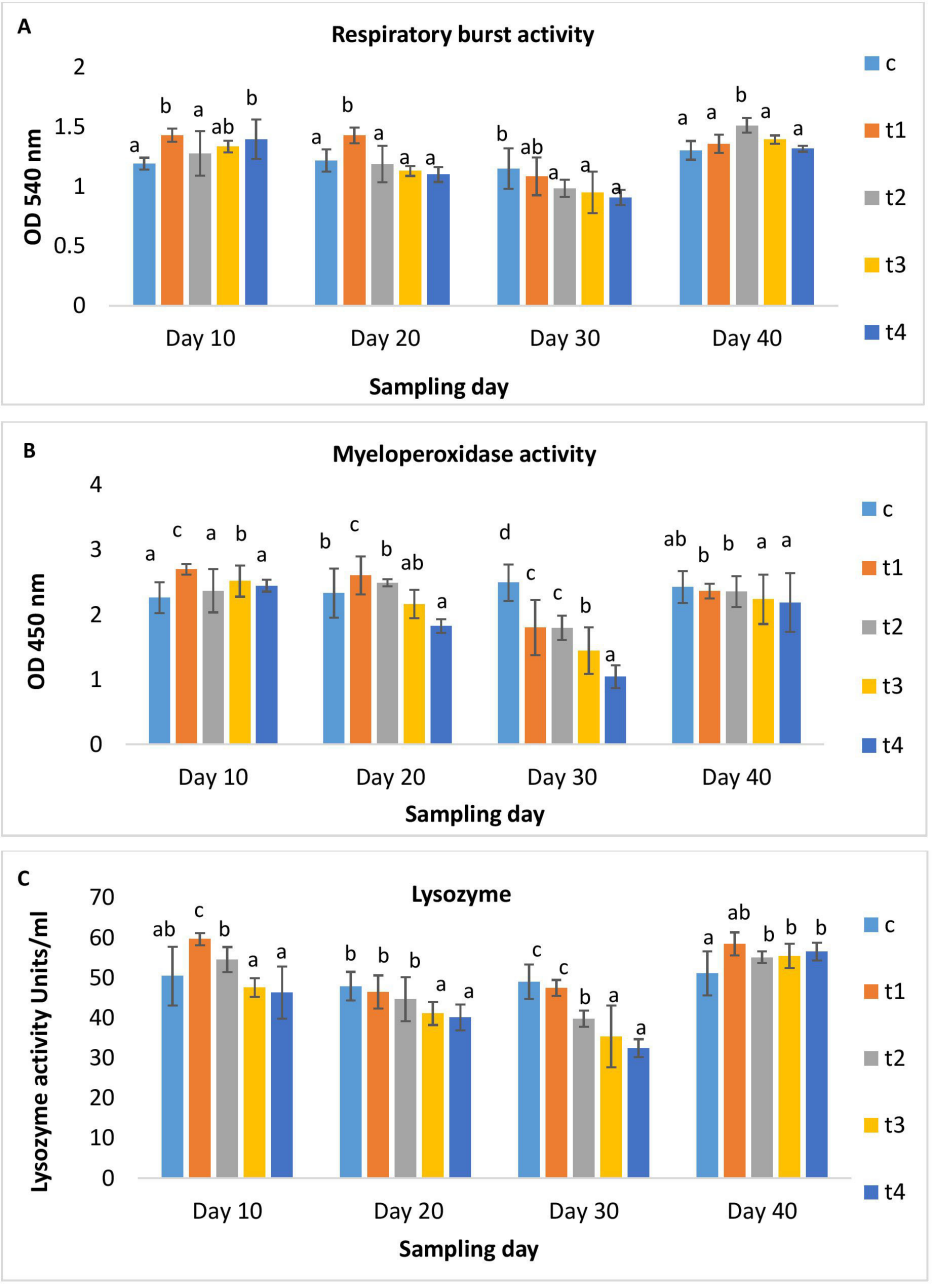
**(A)** Respiratory burst activity, **(B)** Myeloperoxidase activity and **(C)** Lysozyme activity of *H*. *fossilis* in the control and treated groups. Error bars with different alphabets on a specific day indicated significant differences (*p* < 0.05) among groups. OD, Optical density.

The body weight of the OTC-fed (1x-10x) and control groups did not differ significantly during the feeding trial. Both the control and treatment groups documented 100% survival. Only the higher-dosed groups (5x and 10x) showed distinct behaviour abnormalities. The results of the non-specific immune parameters such as RBA, MPO, and lysozyme at different time points are shown in **Figures 1A–C**. On the 10th and 20th day, the RBA and MPO increased significantly (*p*<0.05) in the 1x group compared to the control and then reduced on the 30th day of OTC administration. Further, the other OTC doses insignificantly reduced the RBA, MPO, and lysozyme on the 30^th^ day of administration. The lysozyme was elevated on the 10^th^ day in the 1x and 3x groups, while in other OTC groups, it decreased (**Figure 1C**). On day 10 post-administration, elevated RBA, MPO and lysozyme levels were observed in comparison to the control.

### Biochemical parameters

**Figure 2:**
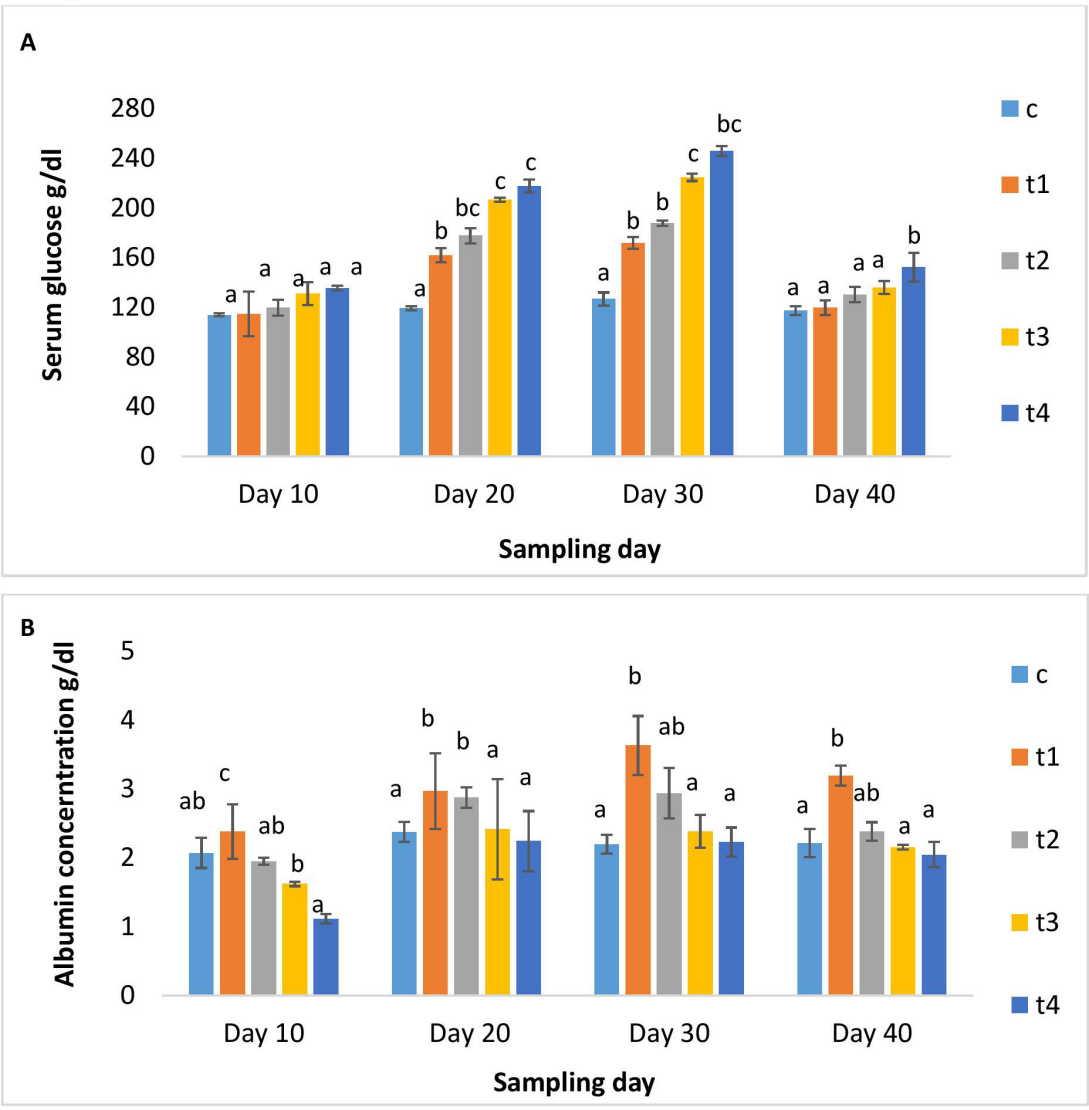
**(A)** Glucose and **(B)** albumin content of *H*. *fossilis* in the control and treated groups. Error bars with different alphabets on a specific day indicated significant differences (*p* < 0.05) among groups. Experimental period: Day 10, 20 and 30: OTC dosing period. Day 40: Day 10 post-OTC-dosing.

**Figure 3:**
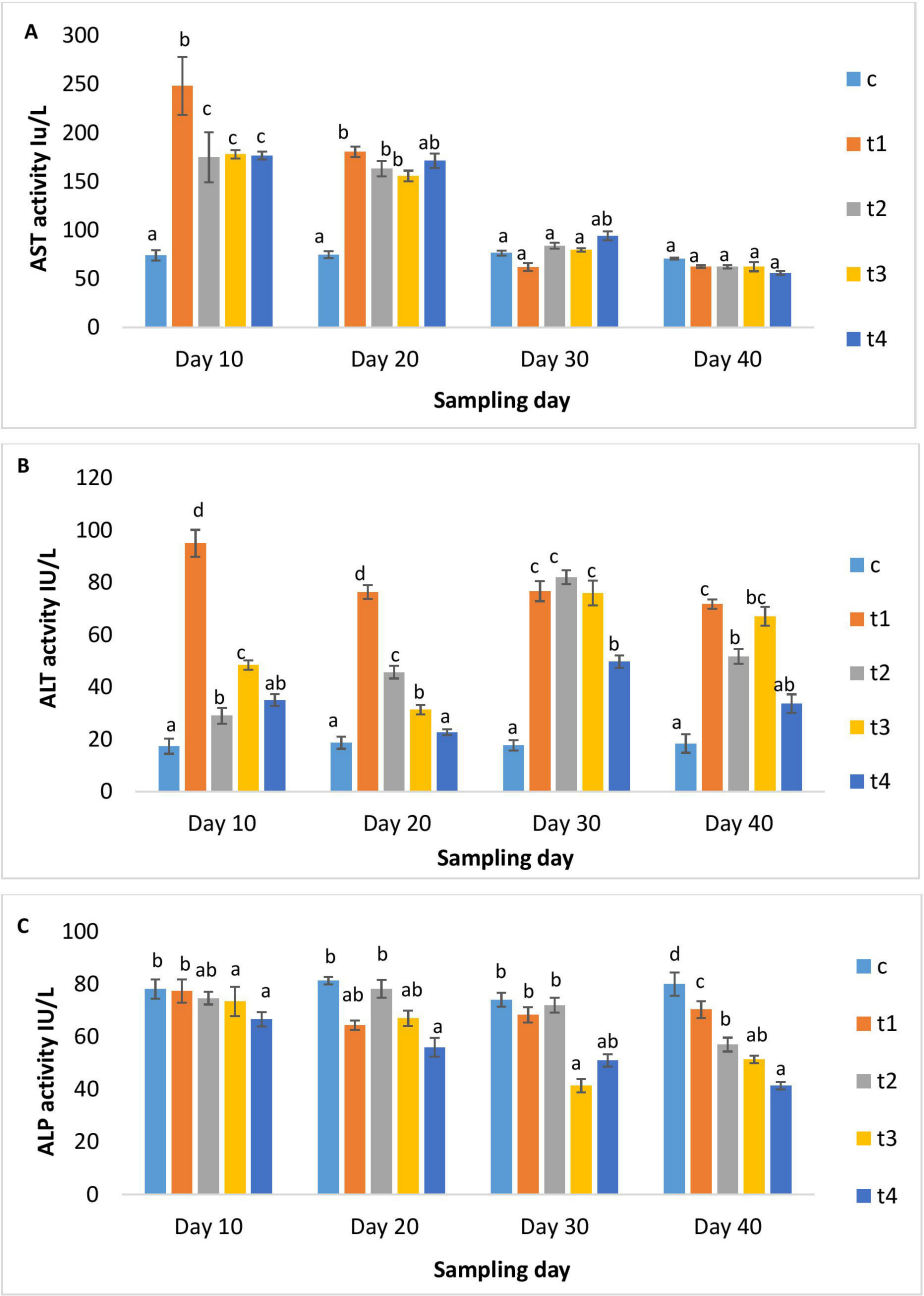
**(A)** AST, **(B)** ALT and **(C)** ALP activity of *H*. *fossilis* in the control and treated groups. Error bars with different alphabets on a specific day indicated significant differences (*p* < 0.05) among groups. Experimental period: Day 10, 20 and 30: OTC dosing period. Day 40: Day 10 post-OTC-dosing.

**Figures 2A, B** illustrated serum glucose and albumin levels in different treatment and control groups. As the dose and duration of administration increased, serum glucose increased significantly in the treatment groups. The higher-dosed groups (5x and 10x) showed elevated serum glucose. On day 10 post-administration, its level decreased significantly in all groups except for the 10x group. The serum albumin levels in the 1x group significantly rose over the periods of 10, 20, and 30 days, followed by a minor decrease upon discontinuation of the dosage. While in other OTC groups, serum albumin was significantly lower than in the 1x group on all days of observation (**Figure 2B**). A significant rise in AST levels in all treatment groups on day 10 was observed, which decreased with time (**Figure 3A**), The ALT levels significantly increased in all treatment groups with dose and time compared to the control (**Figure 3B**). A dose-dependent decrease in ALP activity was observed on all days compared to the control, with the maximum reduction in the 10x group even on day 10 post-administration (**Figure 3C**).

### Oxidative stress biomarkers

**Figure 4:**
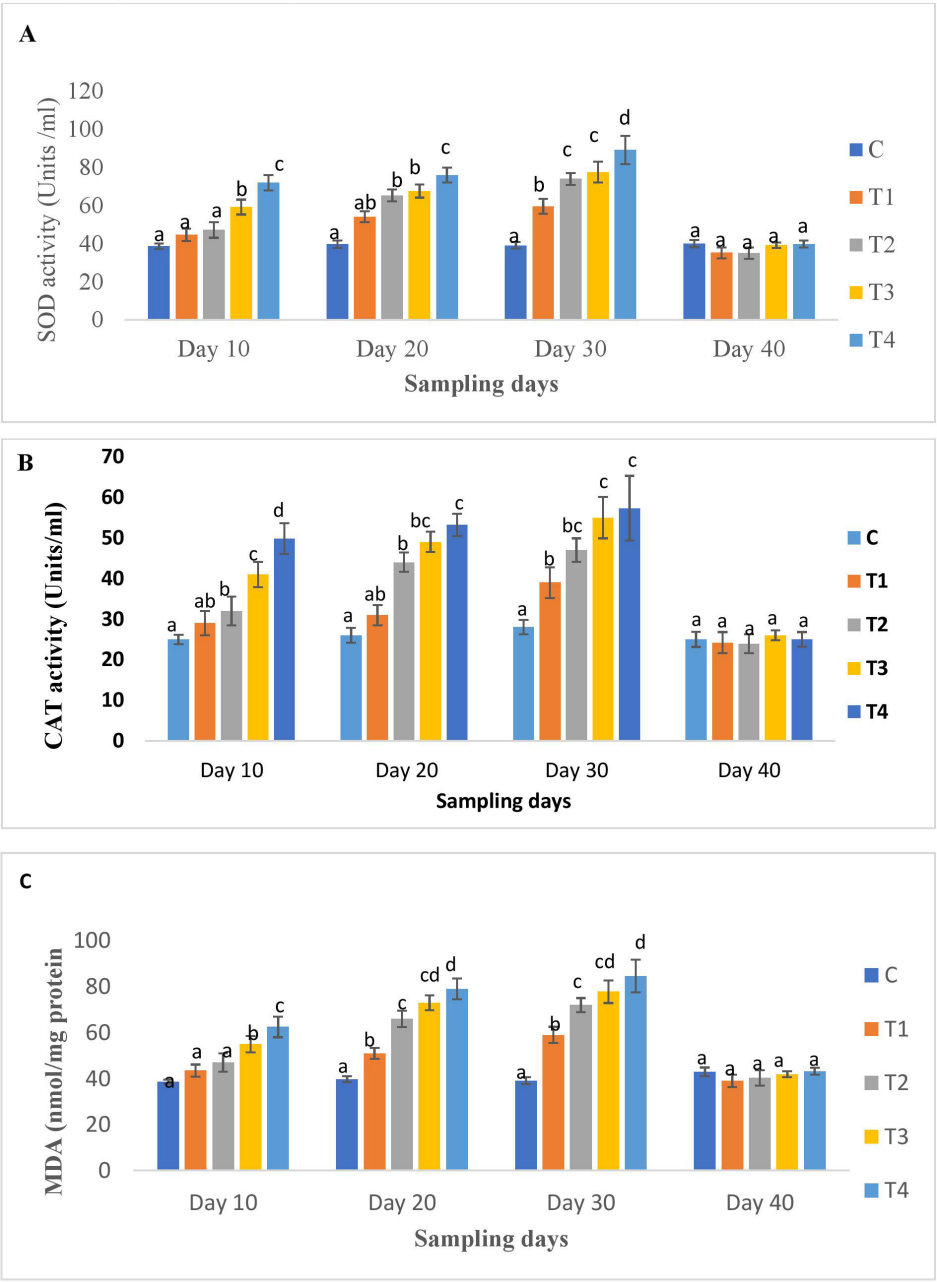
**(A)** SOD, **(B)** CAT and **(C)** MDA activity of *H*. *fossilis* in the control and treated groups. Error bars with different alphabets on a specific day indicated significant differences (*p* < 0.05) among groups. Experimental period: Day 10, 20 and 30: OTC dosing period. Day 40: Day 10 post-OTC-dosing.

The results of the oxidative stress biomarkers in the liver tissues at different time points are shown in **Figures 4A–C**. A significant dose-dependent increase in SOD, CAT, and MDA levels were noted on all days except day 40 of OTC administration compared to the control. The higher dose groups exhibited the highest activity. On day 40 (post-administration), their levels decreased significantly in all groups and were comparable to the control (**Figures 4A–C**).

### Residues in muscle

The dose- and time-dependent residue accumulation and depletion in the edible tissue of the OTC-fed *H. fossilis* are presented in **Table 3**. Among the treatment groups, the 10x group recorded the highest residues (423.3 ± 3.38 µg/kg), surpassing the maximum residual limit (MRL), in the edible flesh on day 30 of administration. However, in the therapeutic dose group, the residues remained below the MRL, measuring 79.5 ± 2.43 µg/kg on day 30 of administration. After 10 days of dose cessation, the residues were reduced below the MRL of 100 µg/kg in 1x and 3x groups, while in other groups the levels were high (**Table 3**).

**Table 3:** Oxytetracycline (OTC) residues in the edible muscle tissues of *H. fossilis* as assessed by LC-MS/MS in different treatment groups.

Treatment groups	OTC residue levels (µg/kg)			
	10^th^ Day	20^th^ Day	30^th^ Day	40^th^ Day
Control (Basal diet)	ND	ND	ND	ND
T1 (80 mg/kg biomass/day)	19.70 ± 0.73^a^	42.50 ± 1.33^b^	79.50 ± 2.43^c^	24.00 ± 0.92^a^
T2 (240 mg/kg biomass/day)	45.40 ± 1.32^a^	93.00 ± 2.22^b^	238.00 ± 4.03^c^	86.00 ± 1.04^b^
T3 (400 mg/kg biomass/day)	78.30 ± 1.05^a^	142.00 ± 2.03^b^	289.30 ± 2.84^c^	112.00 ± 2.06^b^
T4 (800 mg/kg biomass/day)	175.00 ± 2.14^a^	219.30 ± 2.14^b^	423.30 ± 3.38^c^	243.00 ± 2.43^b^

Values within a row with different superscripts (a-c) differed significantly (p< 0.05) ND, Not detected. Experimental period: Day 10, 20 and 30: OTC dosing period. Day 40: Day 10 post-OTC-dosing.

### Liver histopathology

**Figure 5:**
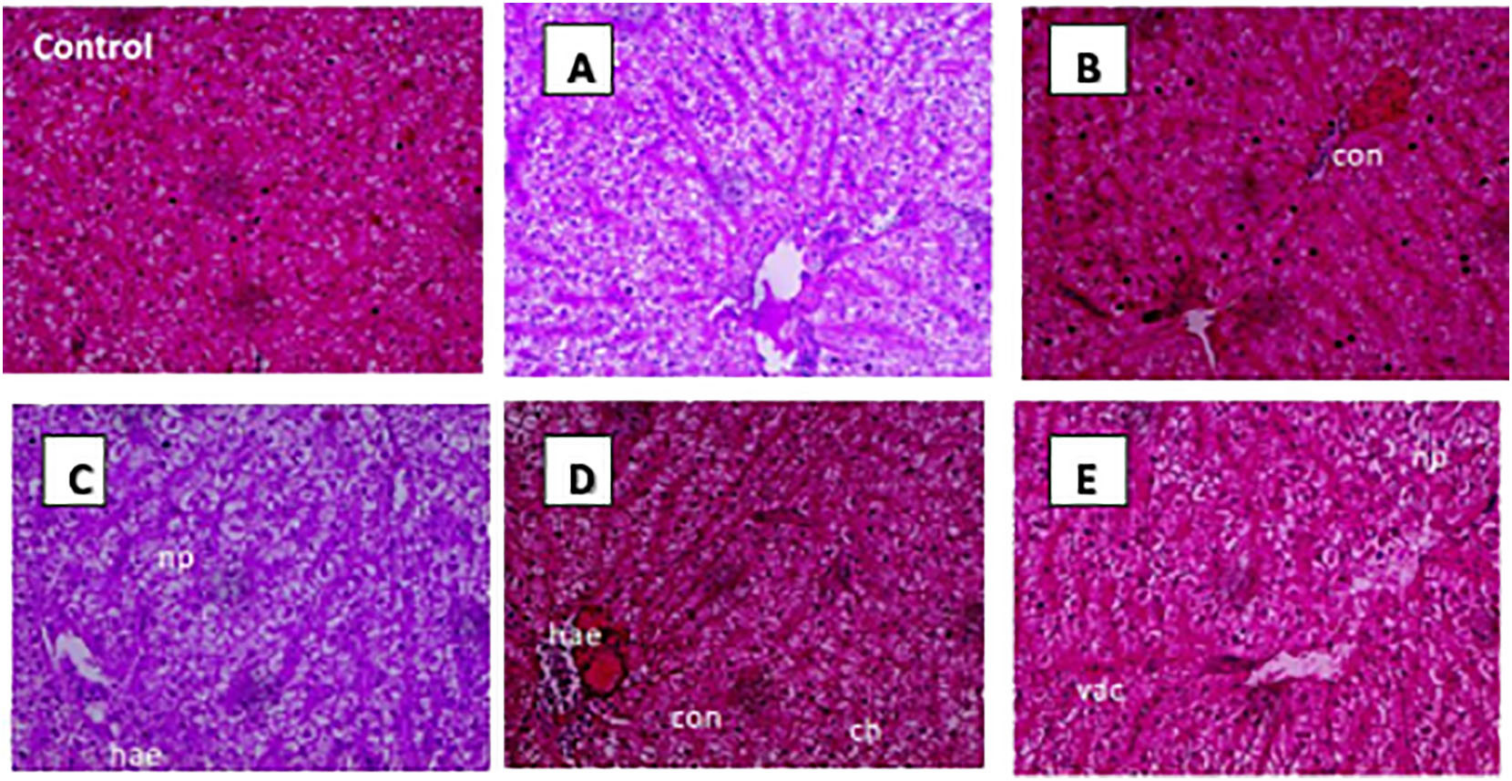
Histopathological changes in *H*. *fossilis* liver tissues of treated groups: Control with normal hepatocytes and sinusoidal architecture; **(A)** T1 treatment group with 1× OTC in diet for 30 days showing almost normal architecture, **(B)** T2 treatment group with 3× OTC for 30 days showing extensive congested hepatic sinusoids; **(C)** T3 treatment group with 5× OTC showing nuclear pyknosis and haemorrhages; **(D)** T4 treatment group with 10× OTC showing diffuse haemorrhage, and cellular hypertrophy; **(E)** T4 treatment group with 10× OTC showing vacuolation, and haemorrhage on the 30^th^ day (hae, haemorrhage; np, nuclear pyknosis; con, congestion; vac, vacuolation; ch,cellular hypertrophy).

The control group exhibited healthy and normal histological features, characterized by intact sinusoidal architecture and hepatic cells with nuclei throughout the experimental trial. The liver structure was severely impacted by OTC administration at the highest dose across all time durations. It displayed significant changes such as haemorrhage congestion, nuclear pyknosis, cellular hypertrophy, and vacuolation. While in other groups, the changes were minimal (**Figure 5**).

## Discussion

OTC is a widely used and highly preferred tetracycline-based antibiotic that is effective against a wide range of bacteria that can infect fish and is legal to use in aquaculture around the world (5, 16, 29). The most common method for administering antibiotics to treat and manage bacterial diseases in aquaculture is through medicated feed at a therapeutic or recommended dose (7, 11, 30). In this study, the tested bacterial strains were infective and isolated from sick fish with no documented evidence of OTC use. The range of OTC’s MIC values (0.5-8.0 µg/mL) against the examined pathogens was low, corroborating the findings of Gaunt et al. (31) in *Aeromonas* spp., *Edwardsiella* spp. and *Streptococcus* spp., with MIC values less than 1.0 regardless of their isolation location and times. These observations on low MIC can help predict the likelihood of a successful therapeutic intervention (32, 33). Before approval, the antibiotics are tested for their effectiveness, biosafety, impact on the environment, and ability to leave residues in the muscle (9, 30, 34, 35). As there are no effective guidelines on the use of OTC on tropical Indian fish, the current work assessed the safety of its oral supplementation at the therapeutic dose as well as up to ten times the therapeutic dose and its effect on several parameters of *H. fossilis.* Dietary supplementation did not cause any mortalities in treatment groups, thus proving its safety even at the highest dose and extended feeding regime. These results were comparable with previous studies recorded in rohu (12), snub-nosed pompano (13), Nile tilapia (14), striped catfish (15) and mrigal (16). However, the results revealed growth retardation in the treatment groups. Earlier reports suggested adverse impacts of OTC on fish possibly due to impaired nutritional absorption and assimilation, internal vital organ injury, disrupted metabolic functions and gastrointestinal microbiome dysbacteriosis (35, 36). Also, no significant differences in growth in rohu and mrigal upon feeding graded doses of OTC for up to 30 days were documented (12, 16). On the contrary, some reports categorized OTC as a growth promoter in striped catfish (15), Nile tilapia (37), and channel catfish (38).

It is reported that OTC-supplemented feed either enhances or reduces the non-specific immune responses (39). The diminished activities of the tested innate immune parameters like RBA, MPO, and lysozyme indicated immunosuppression, particularly at the higher OTC doses (5x-10x groups), supporting reports of Hand et al. (40). However, in agreement with the present results, the innate immunity indices of sea bream increased upon OTC administration at 75 mg/kg biomass/day for 10 days, which was retained up to three weeks after cessation of OTC dosing and then returned to normal levels (41) and the therapeutic dose demarcated significant enhancement in non-specific immunity parameters such as RBA and MPO. The present results on compromised immunity during the feeding trials suggest that the impact on immune responses is species-specific as the fish immunity varies with rearing conditions, fish species, antimicrobial dose, water temperature, route of delivery, and other conditions that affect drug absorption (36).

With the gradual increase in dose and duration of OTC administration, serum glucose increased, suggesting an aberrant metabolic activity and, consequently, stress-related events in fish similar to earlier studies (12, 15). The albumin level increased in the lower dose groups but declined in the higher dose groups, indicating biphasic activities and altered physiological status. The current finding reported reduced AST activity after an initial hike in the treatment groups, while ALT activity increased significantly in all treatment groups. The results indicated liver injury and dysfunction (42, 43), attributable to oxidative stress. Furthermore, elevated ALT levels in serum are suggestive of hepatitis arising from the toxicity of dietary OTC in *H. fossilis* at higher doses and the extensive burden on hepatic integrity. Concurrent with the present findings, Manna et al. (15) reported serum AST and ALT upsurge in catfish*-*fed graded doses (1x - 10x) of OTC. In another study, biphasic activities of AST and ALT were documented, with the highest levels in lower OTC-dosed fish than at the higher OTC-dosed fish (44). The increased concentration of these liver functional enzymes at lower doses and initial treatment times and then decreased concentration at higher doses and prolonged treatment may hypothesized to be the responses of *H. fossilis* is more likely due to terminal stages of feeding graded doses of OTC. Serum ALP was lower in fish-fed-graded OTC doses than in the control. The ALP levels decreased significantly in the 5x and 10x groups on the 30th day of administration, which indicated impaired phosphorus metabolism in OTC-fed catfish. With dose discontinuation, the levels of AST almost stabilized. On the other hand, the ALT and ALP levels were either higher or lower, indicating severe liver injury.

The SOD and CAT are vital for the detoxification of harmful reactive oxygen species, protecting cells, and maintaining homeostasis (45, 46). The MDA levels are considered an indicator of lipid peroxidation caused by oxidative stress and induced by exposure of fish to nutrients, pollutants, antimicrobials, etc. (45, 46). The present work documented a significant dose- and time-dependent increase in SOD, CAT, and MDA in comparison to control, revealing an impairment in the antioxidant systems of *H. fossilis* as a result of oxidative stress. Comparable results were obtained in pearl gentian grouper (10) and tilapia (14, 35) during the oral supplementation of OTC at varied doses. These results intricated an interplay between oxidative stress and drug toxicity on the vulnerability of aquatic organisms and highlighted the importance of monitoring antioxidant defences as a crucial aspect of fish health assessment.

While temperate fish species have well-documented data on residues (30, 39, 47), little is known about the depletion of OTC in tropical fish species. In recent years, reports on OTC residues in tropical fish species, including rohu (12), striped catfish (15), Nile tilapia (14), mrigal (16), and snub-nosed pompano (13) are expanding. The data on antibiotic residues in edible muscle tissues of each fish species is critical for the preparation of food safety guidelines (13), and comprehensive documentation of food safety rules for medication withdrawal times in aquaculture is required. The regulatory firms have set the MRL for OTC in edible fish flesh as 200 µg/kg (48) and 100 µg/kg (49). In this study, the residue levels in the edible flesh of *H. fossilis* were found to be significantly different based on dose and duration of administration. The therapeutic dose group documented residues below the MRL, indicating their compliance with the regulatory guidelines (48, 49). In contrast, OTC residues were above the MRL in higher-dosed groups. The residue accretion in various tissues led to metabolic stress, impaired liver function, oxidative stress and alterations in the liver histoarchitecture. These findings suggested that the use of a therapeutic dose of OTC even for an extended duration poses minimal threat to catfish health or human consumers.

The liver is the best organ for histopathological investigations to assess the drug effects (35, 50), as it is imperative for fish growth and metabolism (51). The histoarchitecture of the liver was severely impacted by OTC dose and duration and was directly correlated with the results of oxidative stress and liver function biomarkers. The hepatic parenchyma of the higher-dosed groups showed severe congestion, haemorrhage, and vacuolation, corroborating several earlier studies that reported hepatic vacuolization upon OTC administration in varied fish species (13, 15, 50–53). Vacuole formation may be a consequence of severe damage and degeneration as the liver is the main detoxification organ. It is, therefore, important to take these hepatotoxic effects into account, particularly if the fish are fed higher doses of antibiotics or longer durations.

## Conclusion

In this study, OTC did not enhance fish growth, even when administered over an extended period. Notably, the therapeutic OTC dose (80 mg/kg biomass/day) did not negatively impact the non-specific immunological indices, enzymatic activities, stress biomarkers and residue accretion. However, administering OTC at higher doses (5x and 10x) had significant adverse effects on innate immunity, biochemical and oxidative stress markers, and liver histopathology. Therefore, to maintain optimal immunological and physiological indices in *H. fossilis*, a therapeutic dose of 80 mg/kg biomass/day may be adhered to for the treatment of bacterial infections. Additionally, higher doses led to a significant accumulation of OTC residues in the edible muscle of *H. fossilis*, which persisted even on day 10 of discontinuation of administration. Considering the negative effects of antibiotics, future research should focus on alternatives or other next-generation antibiotics to better control bacterial infections. This strategy will minimize the impact of antibiotics on cultured fish and other aquatic flora and fauna, as well as mitigate the issue of antimicrobial resistance and public health.

## Data Availability

The original contributions presented in the study are included in the article/supplementary material. Further inquiries can be directed to the corresponding authors.
